# Performance of an active fixation bipolar left ventricular lead vs passive fixation quadripolar leads in cardiac resynchronization therapy, a randomized trial

**DOI:** 10.1002/joa3.12450

**Published:** 2020-11-08

**Authors:** Havard Keilegavlen, Peter Schuster, Thomas Hovstad, Svein Faerestrand

**Affiliations:** ^1^ Department of Heart Disease Haukeland University Hospital Bergen Norway; ^2^ Department of Clinical Science University of Bergen Bergen Norway

**Keywords:** biventricular pacemaker, cardiac resynchronization therapy, heart failure

## Abstract

**Background:**

Usage of active fixation bipolar left ventricular (LV) leads represents an alternative approach to the more commonly used passive fixation quadripolar leads in cardiac resynchronization therapy (CRT). We compared a bipolar LV lead with a side screw for active fixation and passive fixation quadripolar LV leads.

**Methods:**

Sixty‐two patients were before CRT implantations randomly allocated to receive a bipolar (n = 31) or quadripolar (n = 31) LV leads. Speckle‐tracking radial strain echocardiography was used to define the LV segment with latest mechanical activation as the target LV segment. The electrophysiological measurements and the capability to obtain a proximal position in a coronary vein placed over the target segment were assessed.

**Results:**

Upon implantation, the quadripolar lead demonstrated a lower pacing capture threshold than the bipolar lead, but at follow‐up, there was no difference. There were no differences in the LV lead implant times or radiation doses. The success rate in reaching the target location was not significantly different between the two LV leads.

**Conclusions:**

The pacing capture thresholds were low, with no significant difference between active fixation bipolar leads and quadripolar leads. Active fixation leads did not promote a more proximal location of the stimulating electrode or a higher grade of concordance to the target segment than passive fixation leads.

## Graphical abstract

An active fixation bipolar left ventricular pacemaker lead was compared to passive fixation quadripolar leads in a randomized trial. There were no differences between the types of leads in pacing capture thresholds, procedure times, or success rate to reach the target location.

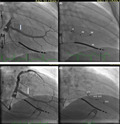

## 1 INTRODUCTION

Cardiac resynchronization therapy (CRT) reduces heart failure symptoms and improves clinical outcomes in selected patients with broad QRS complex.^1^, ^2^ This treatment has proven beneficial, with a reduction in the mortality and hospitalization rates when combined with medical therapy. However, a significant fraction of patients do not experience improvements in symptoms or cardiac function.^3^ A nonoptimal position of the left ventricular (LV) lead is a major reason for an inferior response to CRT.^4^, ^5^ Placement of the LV lead in a segment remote from the region with the latest mechanical activation or in a segment with a myocardial scar predicts a high risk for nonresponse. Echocardiographic speckle‐tracking two‐dimensional (2D) radial strain imaging has the ability to identify the LV segment with the latest mechanical activation. LV lead implantation guided by this robust echocardiographic method has been shown to augment the clinical outcomes of CRT compared with those of unguided LV lead placement.^6^, ^7^ The optimal location for LV pacing may be different from the best position for lead stability and may be compromised to achieve a stable lead position with a low risk of lead dislodgement. Available quadripolar LV leads provide multiple options of different pacing vector and are particularly useful for eliminating postoperative phrenic nerve stimulation (PNS) by reprogramming lead configuration.^8^, ^9^ Active fixation mechanisms of LV leads facilitate stable lead positions in a wide range of venous anatomies.^10^ The aim of the current study was to compare a bipolar LV lead with a side helix for active fixation and a quadripolar LV lead with passive fixation regarding the electrophysiological performance, the stability, and the ability to reach the target position.

## 2 METHODS

### 2.1 Study design

In this prospective, randomized, single‐center trial, patients with symptomatic heart failure and an indication for CRT implantation in accordance with current guidelines were included. The study was approved by the regional committee for medical and health research ethics (Reference 2015/1507), and all patients gave their written informed consent. The patients were blinded and randomly assigned to receive either an active fixation lead or a quadripolar passive fixation lead. For patients randomized to receive a quadripolar lead, the operators were free to choose between three different shapes. Prior to randomization, the patients were stratified into two cohorts based on whether they received a CRT device either with a defibrillator (CRT‐D) or without a defibrillator (CRT‐P). The decision of implanting a CRT‐D or a CRT‐P was done individually based on etiology of the heart failure and the patient's comorbidity.

### 2.2 Patient population

Between February 2016 and November 2017, 62 patients were included and randomized. The inclusion criteria, which were based on current guidelines, were symptomatic heart failure; New York Heart Association (NYHA) functional class II or III or ambulant class IV; LV ejection fraction ≤35%; and left bundle branch block (LBBB) with a QRS duration ≥120 ms or non‐LBBB with a QRS duration ≥150 ms The baseline clinical characteristics and comorbidities of the patients are described in Table 1. No significant differences were found between the two patient groups with respect to sex, QRS duration, LV ejection fraction, NYHA functional class, medication, and comorbidities. The average NYHA functional class was 2.7 in both patient groups.

**TABLE 1:** Baseline characteristics

	Active fixated lead (n = 31)	Quadripolar lead (n = 30)	*P*‐value
Female sex, n (%)	11 (35)	6 (20)	.18
Age, years	71.5 ± 13	72.2 ± 10	.82
Left ventricular ejection fraction, %	24.4 ± 6	27.0 ± 5	.07
Left bundle branch block, n (%)	29 (94)	29 (94)	.58
QRS duration, ms	165 ± 19	162 ± 18	.56
PR time, ms	193 ± 32	191 ± 29	.79
NYHA II, n (%)	11 (35)	11 (37)	.93
NYHA III or IV, n (%)	20 (65)	19 (63)	.93
Ischemic cardiomyopathy, n (%)	17 (55)	20 (67)	.35
Hypertension, n (%)	16 (52)	15 (50)	.90
Diabetes, n (%)	5 (16)	9 (30)	.20
Permanent atrial fibrillation, n (%)	4 (13)	6 (20)	.46
Paroxysmal atrial fibrillation, n (%)	7 (23)	10 (33)	.36
Smoker, n (%)	2 (6)	4 (13)	.38
ACE inhibitors, n (%)	31 (100)	30 (100)	1.00
Betablockers, n (%)	29 (94)	30 (100)	.16
Aldosterone inhibitors, n (%)	11 (35)	12 (40)	.72
Diuretics, n (%)	17 (55)	19 (63)	.51
CRT‐D, n (%)	20 (65)	20 (67)	.86

### 2.3 Echocardiographic imaging

The LV ejection fraction was measured by echocardiography using the biplane modified Simpson's method (GE Vivid E9, Vingmed Ultrasound, Horten, Norway). Transthoracic echocardiography with 2D speckle‐tracking radial strain (ST‐RS) measurements of the LV was performed prior to the implantation procedures. All images were processed offline (EchoPac 202 GE Medical System, Horten, Norway). Intraventricular LV dyssynchrony was determinated using ST‐RS echocardiography from 2D images in a mid‐LV parasternal short‐axis view with a frame rate ≥50 Hz. Time‐strain curves were computed for the different LV segments. Left ventricular segments with a strain rate <10% were excluded because this finding was considered to indicate a high level of transmural scarring.^11^, ^12^ The time from Q‐wave onset on the electrocardiogram to the maximal radial strain in the anterior, lateral, and posterior LV segments was calculated as an average of five consecutive cardiac cycles. The latest contracting LV segments were identified for the anterior, lateral, and posterior LV segments. If the latest contraction of two of the LV segments was separated by ≤10 ms, the LV segment located between them was assigned the latest one. Based on this model, 5 LV segments were defined; thus, the target LV segment for LV lead placement could be identified as the anterior, anterolateral, lateral, posterolateral, or posterior segment. The LV segments next to the target segment were classified as adjacent LV segments, and other segments were classified as remote LV segments.^13^

### 2.4 Cardiac resynchronization therapy device implantation

**FIGURE 1:**
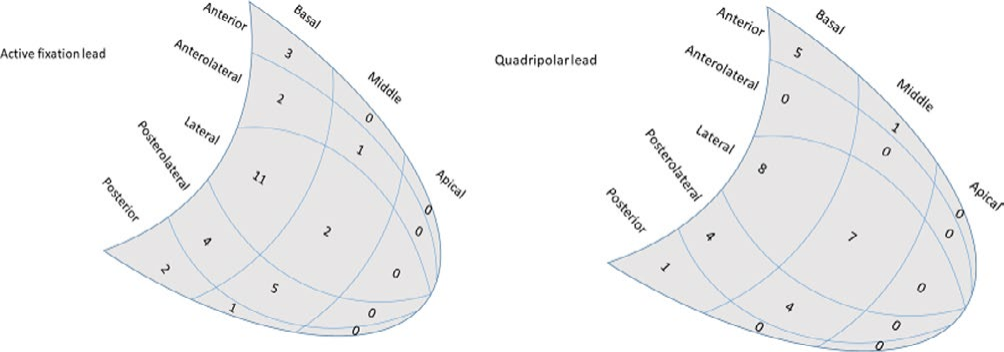
Location and number of selected stimulating electrodes in different left ventricular segments in the active fixation lead group and in the quadripolar lead group

The devices were implanted under local anesthesia. The right atrial (RA) lead was fixated in the appendage of the right atrium, and the right ventricular (RV) lead in the apex of the right ventricle. Occlusive contrast venography was performed in a 30‐40° left anterior oblique (LAO) view and in a 30° right anterior oblique (RAO) view once the coronary sinus (CS) was cannulated. A selective venogram in a 30° RAO view was performed for the accurate measurement of the LV long‐axis distance, which was divided into three equal segments: basal, middle, and apical (Figure 1). From the venogram in the LAO view, the left ventricle was divided into five equal segments (Figure 1) that corresponded to the five segmental divisions acquired in the preoperative ST‐RS echocardiographic measurement. Thus, the target segment for the LV pacing lead was also located on the venogram in the LAO view. Substantial effort was made to achieve an LV lead position in a vein located in the target segment with the latest contraction. If there was no available vein in that segment, a vein located as close as possible was selected for lead placement.

The LV leads were delivered using the over‐the‐wire technique with standard coronary sinus cannulation catheters and a subselection catheter when required. As basal as possible of an LV long‐axis position for the stimulating electrode was preferred. The measurements of the pacing capture threshold (PCT) and the occurrence of phrenic nerve stimulation (PNS) were recorded. For the active fixation lead, a J‐shaped stylet was inserted to press the helix toward the vessel wall. The lead was then fastened by clockwise rotation. The lead fixation was verified by pushing and pulling the lead during observation of lead movement using fluoroscopic imaging. If repositioning of the lead was needed, counterclockwise rotation was performed to free the lead helix from the vein wall. The R‐wave, pacing impedance, and electrical delays as well as the Q‐LVsense, RVsense‐LVsense, and RVpace‐LVsense were recorded from a pacemaker system analyzer (Model 2090, Medtronic, Minneapolis. MN, USA) before removing the catheters. The leads were connected to a CRT‐D or a CRT‐P generator. The devices used were CRT defibrillators (CRT‐D, Medtronic, Minneapolis, MN, USA) in 66% of the patients and CRT pacemakers (CRT‐P, Abbot, Lake Bluff IL, USA) in 34% of the patients.

### 2.5 Lead characteristics

**FIGURE 2:**
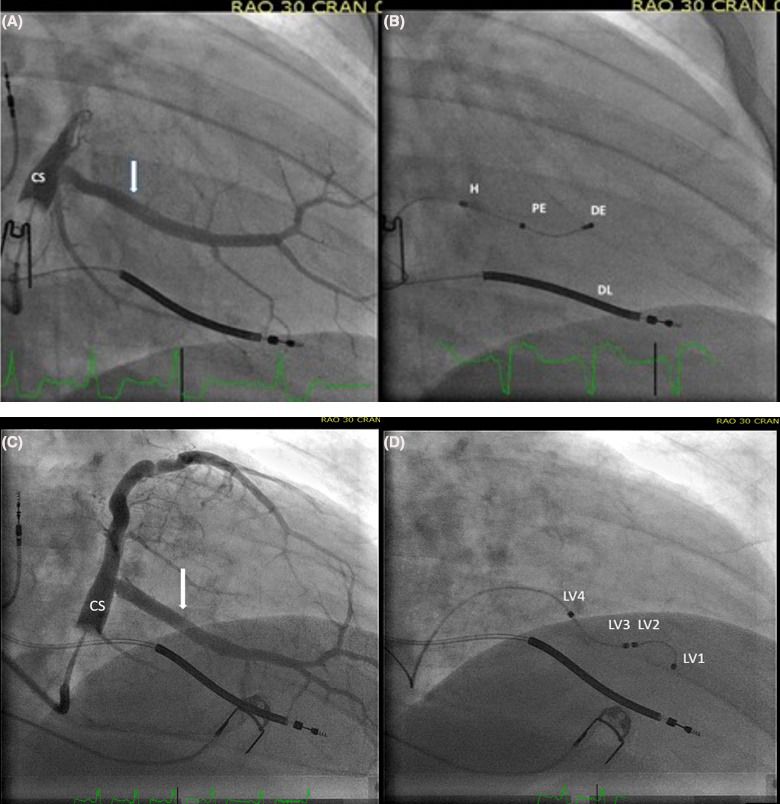
Right anterior oblique fluoroscopic views of two patients with an active fixation bipolar lead (A + B) and passive fixation quadripolar lead (C + D). On the coronary sinus (CS) venograms (A + C) the arrows indicate the target veins in lateral side branches from CS. The target vein is located in the target left ventricular segment determined from speckle tracking echocardiography. B: The final lead placement of an active fixation bipolar lead. The helix (H) is fixated proximal in the vein. The proximal electrode (PE) is located in a basal third left ventricular long‐axis position, and is used as the stimulating cathodal electrode. The distal electrode (DE) is in the mid third left ventricular long‐axis segment. The high voltage right ventricular defibrillator lead (DL) is located close to the apex of the right ventricle. D: The final lead placement of a quadripolar lead. The distal end (LV1) is wedged into a small side branch. The proximal electrode (LV4) is used as the stimulating cathodal electrode

**FIGURE 3:**
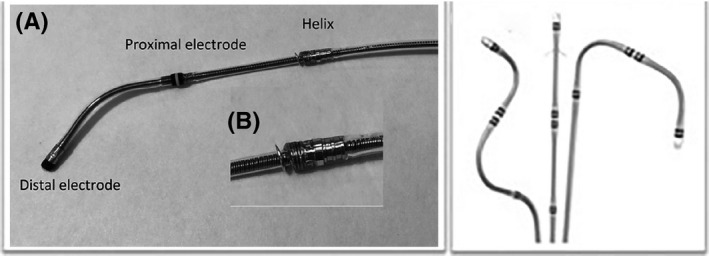
A, The bipolar lead with distal angled shape has an exposed side screw for fixation located 15 mm proximal to the proximal electrode. The electrode separation is 21.0 mm. The maximum lead body diameter is 3.9 Fr. B, Demonstrates a close range view of the exposed side screw. C, The quadripolar leads: An S‐shaped lead, a straight lead with tines and a dual bend lead. The distances between the electrodes are 21 mm (LV1‐LV2), 1.*3 mm (LV2‐LV3), and 21 mm (LV3‐LV4). The maximum lead body diameter is 5.3 Fr A + B: Photo by the author. C: Photo from the manufacturer*

The active fixation lead was a soft bipolar steroid‐eluting lead (Attain Stability model 20066/4796, Medtronic Inc, Minneapolis, MN, USA). The lead body was 3.9 French (Fr) proximal and 3.4 Fr distal (Figures 2 and 3). The electrode separation was 21 mm. Proximal to the ring electrode was a side screw. Longitudinal movements of the lead without torqueing did not engage the screw. The screw was also designed to elongate along the length of the lead body and to detach it from the vein wall when the traction force was increased to approximately 0.11 kilograms. The quadripolar leads (Figures 2 and 3), which were attained from the same vendor, had a diameter of 5.3 Fr proximal and 3.9 Fr distal. A dual bend lead, an S‐shaped lead and a straight lead with tines were available. The dual bend lead was used in 19 patients (63%), the S‐shaped lead in 10 of the patients (33%) and the straight lead in one patient (3%).

### 2.6 Programming the device

Atrioventricular (AV) and interventricular adjustments were based on an automatic algorithm (adaptive CRT, Medtronic, Minnesota Inc, MN, USA) for patients with a CRT‐D generator. The CRT‐P devices were programmed without any LV off‐set, and the sensed‐AV‐time was programmed to 120 ms The pacing modus was DDD, lower rate of 50 pulses per minute for those with no sinus node dysfunction. The active fixation bipolar leads were configured as bipolar, LV tip to RV‐coil/RV ring or LV ring to RV coil/RV ring. For the quadripolar leads, the preferred configuration was bipolar L3‐L2, integrated bipolar LV1 to the RV coil/RV ring, LV3 to the RV coil/RV ring, or LV4 to the RV coil/RV ring. A limited number of configurations for the quadripolar lead were selected for an accurate assessment of the location of the stimulating electrode in the LV long‐axis view. The final LV lead position was evaluated. The PCT, R‐wave, and LV lead impedance were measured at the 2‐, 6‐ and 12 month follow‐up (FU) periods.

### 2.7 Statistical analysis

Analyses were conducted according to the intention‐to‐treat concept. Statistical analysis was performed by IBM SPSS Statistics for Windows, version 24.0 (IBM Corp., Armonk, NY, USA). Continuous variables are presented as the mean ± SD. Categorical variables are shown as frequencies and percentages. Differences were determined using Student's *t*‐tests for continuous variables, the chi‐square test for ordinal variables, or Fisher'exact test for categorical variables. We used histograms and Q‐Q plots to evaluate the normality of the continuous variables. A *P*‐value of < .05 was considered statistically significant. For sample size calculation, a 20% difference in the proportion of concordant LV lead placement, fluoroscopic distances, lead impedance measurements, and PCTs were estimated. Descriptive data from a previous active fixation lead study (10) were used to predict values and standard deviations. Powered at 80%, with a two‐sided alpha value of 0.05 to detect differences, about 60 patients were required for the different analyses. The study was not powered to compute significant differences in infrequent events as lead dislocations.

## 3 RESULTS

Initial successful implantation was obtained in 31 patients (100%) and 30 patients (97%) in the active fixation bipolar group and the quadripolar group, respectively. In 1 patient, the quadripolar lead dislodged repeatedly during implantation, and this could not be avoided by switching to the bipolar active fixation lead. Finally, an alternate thicker bipolar LV lead was implanted successfully. In three patients, LV lead dislodgement occurred, all in the active fixation group. For two of these three patients, the LV lead dislodged some hours after implantation, and the third instance of dislocation was recognized after 2 months. In all three patients, the same lead was repositioned successfully to the same coronary vein. We compared the size of target veins (Table 2). There was no difference in vein size at the active electrode or at the distal electrode. The average vein dimension at the proximal electrode was larger at the qauadripolar lead, corresponding with a more proximal position. During FU, there were no additional instances of reoperation, and there were no cases of device infection. All patients were alive the 12 month FU. Table 2 summarizes the characteristics of the 62 implantation procedures. The locations of the targeted LV segments were anterior (10%), anterolateral (11%), lateral (44%), posterolateral (30%), and posterior (5%). The distribution of the locations of the selected stimulating electrodes for each LV lead is shown in Figure 1. The target LV lead placement, which was defined as a position in a concordant or adjacent LV segment, was achieved in the majority of the patients with no statistically significant differences between the patient groups (Table 3). For both LV lead groups, the selected active electrodes were stimulating the LV from a position close to the distal part of the basal segments in majority of the patients. The proximal electrode of the quadripolar LV lead was closer to the CS than that of the active fixation LV lead. However, there was no statistically significant difference between the active fixation group and the quadripolar group concerning the proximity of the stimulating electrode to the coronary sinus, neither in absolute values nor in distance as a percentage of the distance from the CS to the apex.

**TABLE 2:** Characteristics of the implantation procedures

	Active fixated lead (n = 31)	Quadripolar lead (n = 30)	*P*‐value
Number of veins attempted, n	1.1 ± 0.52	1.29 ± 0.40	.26
Number of fixation attempts, n	1.7 ± 1.5	Not relevant	
Total LV lead implantation time, min	13.2 ± 11	12.2 ± 12	.75
Total procedure time, min	77 ± 22	76 ± 21	.82
Fluoroscopy time, min	15 ± 7	15 ± 10	.68
Fluoroscopy doses, mGY (mGym2)	329 ± 236 (3.0 ± 2.1)	319 ± 426 (3.2 ± 4.3)	.91 (0.85)

Note

Total LV lead implantation time was measured from the start of LV lead insertion and included advancement of the lead to the target site, fixation attempts, repositioning to other locations, electrophysiological measurements, and removal of supporting catheters.

**TABLE 3:** Left ventricular lead positions

	Active fixated lead (n = 31)	Quadripolar lead (n = 30)	*P* value
Lead in the concordant segment, n (%)	12 (39)	19 (63)	.06
Lead in an adjacent segment, n (%)	15 (48)	6 (20)	.02
Lead in a concordant or adjacent segment, n (%)	27 (87)	25 (83)	.69
Lead in a remote segment n (%)	4 (13)	5 (17)	.69
Distance from CS to proximal electrode, mm	32 ± 10	19 ± 15	.00
Distance from CS to distal electrode, mm	51 ± 9	53 ± 13	.51
Distance from CS to active electrode, mm	38 ± 10	35 ± 13	.36
Distance from CS to active electrode as percentage of distance from CS to apex, %	36 ± 11	33 ± 12	.26
Vein size at proximal electrode, Fr	8.1 ± 3.0	10.8 ± 6.2	.04
Vein size at distal electrode, Fr	6.2 ± 2.7	5.3 ± 2.3	.20
Vein size at active electrode, Fr	7.3 ± 2.9	8.3 ± 3.2	.20

Abbreviation: CS, coronary sinus.

The electrical performance was recorded at implantation and at the 2‐, 6‐ and 12 month FU periods (Table 4). For the final selected pacing configurations, the mean PCT for the active fixation lead was higher at implantation but was not significantly different at FU. A PCT < 2.5 V/0.4 ms at implantation was achieved in 100% of patients in both groups. At the 12 month FU, a PCT < 2.5 V/0.4 ms was recorded for 93% of patients in both groups. The PCT for the proximal electrode was significantly higher for the quadripolar lead than for the active fixation lead (2.83 V vs 1.31 V; *P* = .003). For the quadripolar lead, the PCT at the proximal electrode was ≥ 3.5 V for 10 patients (33%); however, for the active fixation lead, the PCT was ≥ 3.5 V only for two patients (6%). At the 12 month FU, nine patients (16%) had at one time or another after discharge from the hospital experienced some kind of discomfort from PNS. Six of those patients (19%) were in the active fixation group, and three (10%) were in the quadripolar group. In all cases, the PNS was resolved by reprogramming the device.

**TABLE 4:** Electrical performance at the selected configurations

	Active fixated lead (n = 31)	Quadripolar lead (n = 30)	*P*‐value
PCT at implantation, V@0.4 ms	1.09 ± 0.48	0.77 ± 0.25	.02
PCT at the 2 month FU, V@0.4 ms	1.23 ± 0.77	1.00 ± 0.62	.21
PCT at the 6 month FU, V@0.4 ms	1.16 ± 0.76	1.02 ± 0.74	.46
PCT at the 12 month FU, V@0.4 ms	1.23 ± 0.75	1.03 ± 0.86	.35
LV lead impedance at implantation, Ohm	539 ± 159	414 ± 94	.00
LV lead impedance at 6 months	561 ± 156	443 ± 96	.01
LV lead impedance at 12 months	545 ± 142	433 ± 97	.04
R wave, mV	17 ± 8	13 ± 8	.03
Q‐LV sense, ms	155 ± 30	154 ± 35	.88
RVsense‐ LVsense, ms	101 ± 26	97 ± 36	.67
RVpace‐ LVsense, ms	142 ± 27	143 ± 31	.94

Abbreviations: PCT, pacing capture threshold; FU, follow‐up; LV, left ventricle; RV, right ventricle; Q‐LV sense, interval from QRS onset to first peak of the LV electrogram.

## 4 DISCUSSION

Implanters of CRT devices are concerned about the acute and chronic lead stability. Much effort has been devoted to developing leads that provide stability and a low PCT, but that have preserved trackability along tortuous veins. LV leads have evolved from unipolar to bipolar and, further, to quadripolar models. Compared to bipolar leads, quadripolar leads provide more available pacing vectors and less PNS. Quadripolar leads have been associated with better clinical response and lower mortality, based on retrospective analyses.^9^, ^14^ In these trials, the LV leads were placed empirical and not targeted, and the final LV lead positions were not assessed. Consequently, it is not possible to conclude if the clinical superiority of quadripolar lead is a consequence of being quadripolar with multiple options for pacing configurations, or if it is because of implantation issues and the final position of the active electrode. According to the subgroup analyses of randomized trials, such as MADIT‐CRT and REVERSE, LV pacing from an apical site is associated with less favorable outcomes and high PNS.^4^, ^15^ However, operators may be tempted to sacrifice a nonapical position to achieve a stable position and low PCT by wedging the lead in a small apical branch. The optimal long‐axis LV lead position is debatable, and the future may show that the optimal long axis position occurs on an individual basis. A nonrandomized multicenter trial that compared active fixation LV leads with quadripolar LV leads reported noninferior clinical outcomes for the active fixation leads.^16^ Our trial is the only randomized clinical trial comparing active fixation LV leads with quadripolar LV leads. At implantation, the PCT was lower in the quadripolar group than in the active fixation group, but the difference decreased later, and there were no significant differences at the 6‐ and 12 month FUs. The pacing impedance was significantly higher (approximately 20%) in the active fixation group than in the quadripolar group and may lead to a moderate increase in battery longevity compared to devices with quadripolar LV leads.

Our hypothesis was that in large veins, an active fixation bipolar LV lead will enable a more proximal position of the stimulating electrode compared to a quadripolar lead. We aimed to achieve a position of the stimulating LV lead electrode as far from the apex as possible. Nevertheless, we did not find any significant difference between the two types of LV leads concerning the proximity of the ultimately selected stimulating electrode to the coronary sinus. Thus, the active fixation lead did not promote a more basal placement of the stimulating electrode. An explanation for this may be that in many cases of quadripolar leads, it was possible to wedge the lead tip in an early side branch to stabilize the stimulation electrode in a basal LV segment. Furthermore, the electrically inactive helix of the active fixation bipolar lead was placed proximal to the proximal electrode, thus prohibiting placement of the proximal electrodes in the vein close to the coronary sinus. The PCT for the proximal electrode was significantly higher for the quadripolar lead than for the active fixation lead (2.84 vs 1.42 V). This may be because of the lower amount of pressure toward the wall for the passive leads than for the active fixation leads in the proximal vein segment. On the contrary, the PCTs for the distal electrodes were lower for the quadripolar leads at implantation. An explanation for this may be that the S‐shape or dual bend shape and the larger body diameter of the quadripolar lead may cause more tension toward the vein wall than that of the distal end of the active fixation lead. Quadripolar leads with active fixation were not available when the current trial was performed, but later, a quadripolar active fixation lead with a similar helix for fixation was approved (Medtronic lead model 4798). In this quadripolar version, the fixation mechanism is located between electrodes 3 and 4, which may potentially improve the lead stability and reduce the PCT for the most proximal electrodes, even in large coronary veins. The LV lead dislodgement rate is low in recent trials with quadripolar leads, and in the Performa Trial, a dislodgement rate of 1.4% was reported.^17^ In the current trial, which was not powered to show differences in the rate of lead dislodgement, there were no dislodgements of the quadripolar leads; however, in the active fixation group, two postoperative dislodgments and one late dislodgment occurred. These three patients were retrospectively evaluated. One patient had a large‐diameter coronary vein (16.5 Fr at the point of helix fixation) and needed four fixation attempts at the primary operation. The other 2 patients with LV lead dislodgement showed no unusual vein‐anatomy and only one fixation attempt was needed initially. The present trial did not prove that adding an active fixation mechanism to bipolar lead makes them more stable than passive fixation quadripolar leads. The new location of the fixation helix between electrodes 3 and 4 in the Medtronic lead model 4798 may potentially further augment the stability of the active fixation lead.^18^ There is an obvious concern about the extractability of active fixation LV lead. Unlike the leads with side lobes of the lead, the Attain Stability is fixated with a side helix constructed to uncoil in response to retractive force. However, the data on extraction safety are limited and this must be taken into account when choosing an LV lead.

In this randomized trial, comparing an active fixation bipolar lead and quadripolar passive fixation leads, no important differences in implantation variables or long‐term electrical performance were found. Furthermore, there were no differences in the ability to reach a proximal concordant or adjacent LV segment for targeted LV stimulation.

### 4.1 Study limitations

The study was a single‐center study with a relatively small sample size. Therefore, the extension of the validity of these results to other centers and implanters is not possible. The clinical findings, as changes in NYHA classification or echocardiographic response, were not compared in the present study.

## CONFLICT OF INTERESTS

The authors have no conflict of interest, financial or otherwise.
